# Prediction of Pathological Subjects Using Genetic Algorithms

**DOI:** 10.1155/2018/6154025

**Published:** 2018-01-29

**Authors:** Murat Sari, Can Tuna

**Affiliations:** Department of Mathematics, Yildiz Technical University, Esenler, Istanbul 34220, Turkey

## Abstract

This paper aims at estimating pathological subjects from a population through various physical information using genetic algorithm (GA). For comparison purposes, *K*-Means (KM) clustering algorithm has also been used for the estimation. Dataset consisting of some physical factors (age, weight, and height) and tibial rotation values was provided from the literature. Tibial rotation types are four groups as RTER, RTIR, LTER, and LTIR. Each tibial rotation group is divided into three types. Narrow (Type 1) and wide (Type 3) angular values were called pathological and normal (Type 2) angular values were called nonpathological. Physical information was used to examine if the tibial rotations of the subjects were pathological. Since the GA starts randomly and walks all solution space, the GA is seen to produce far better results than the KM for clustering and optimizing the tibial rotation data assessments with large number of subjects even though the KM algorithm has similar effect with the GA in clustering with a small number of subjects. These findings are discovered to be very useful for all health workers such as physiotherapists and orthopedists, in which this consequence is expected to help clinicians in organizing proper treatment programs for patients.

## 1. Introduction

Most problems come out in nature are usually represented by mathematical models. To analyze those problems arisen in various fields of science, mathematical modeling has been considered as an important tool. Advent of computers, producing algorithms, and progress in computer programming have made life easier in solving intricate problems of science. This is also the case in problems encountered in biomechanics. To make the best biomechanical decisions, medical prediction plays a very important role for health providers. Specifically, many researchers have concentrated on analysis of the knee motion and many methods were designed to describe the range of motion of it [1]. It is important to predict tibial rotation types of pathologies during daily examination, since there exists a serious link between the tibial motion and various knee injuries [2].

As signified in the literature [3, 4] the knee joint is one of the most complex joints in the musculoskeletal system. To assess the motion of the knee joint, various techniques were suggested to describe the range of motion of the knee joint [1, 5–9]. It is reported that there are limited number of investigations resolving the tibial motion involving the internal and external rotations [4, 10–14]. Note that an excessive internal tibial rotation or a delayed external tibial rotation leads to some knee injuries. Owing to external rotation related to knee extension, excessive internal rotation during the stance phase of walking can postpone the natural external rotation while the knee extends. As underlined by various researchers [2, 15], this situation may cause torsional joint stresses through tibial shaft and by turns lead to knee injury rotation.

Analysis of the tibial motion is usually difficult for medical points of view. Although it is natural to come across attractive studies realized in the literature, the pathological interval of the tibial rotations has not been optimized through the physical information yet. Even though the conventional methods encountered in the assessment of the tibial rotations are still among the attractive topics in the academic society [16–26], researchers have nowadays increased to pay their attention to computational assessment [27, 28] and prediction techniques such as artificial neural networks [4, 14]. Despite recognized advantages of the conventional methods, most of them are suffering from various disadvantages such as high cost, difficulty in use, being time-consuming, and constraints in daily use. In that case, optimization can be recalled as an alternative to the corresponding methods. Various heuristic approaches have been improved in the recent couple of decades that simplify solving optimization problems that had previously serious difficulties. Those approaches include evolutionary computation, tabu search, and particle swarm. Recently, genetic algorithm (GA) and particle swarm optimization (PSO) techniques come out as encouraging approaches for analyzing the optimization problems. Those algorithms are having popularity within academic society as model tools due to their versatility and potentiality to optimize in intricate search spaces. For both GA and PSO approaches, the fundamental issue in implementation lies in the selection of an appropriate objective function. Both approaches are inspired by nature and are shown to be effective solutions to optimization problems. Note that the corresponding algorithms are not a panacea, despite their well-known effectiveness. For some problems, the GA approach is superior to the PSO approach while for some problems the latter approach is superior to the first one [29–32]. The encountered prediction algorithms, like PSO, have great potentiality and in some cases superiorities in analysis of optimization problems. The other one of the two popular methods, the GA, is well-established, flexible, of easy programming, and lower cost, and therefore it is used very often and supplies an alternative approach for information-processing methods. Hence, the aforementioned advantages of the GA sent us to use it in the current study.

This paper predicts pathological subjects from a population through various physical information using the GA. Even though it has been considered for comparison purposes, the KM clustering algorithm has also been developed for the prediction. The developed framework of the GA is successfully applied to medical prediction problems and has achieved superior classification performance to the other competitive counterpart, the KM clustering algorithm. Dataset consisting of some physical factors (age, weight, and height) and tibial rotation values was provided from the work of Sari and Cetiner [4]. Thus, this study discovers potentiality of the two algorithms, the GA and the KM clustering, in predicting the tibial rotation types through the physical factors. To the authors' best knowledge, the GA has not been implemented to predict the tibial rotation type based on the physical information so far. Since the GA is flexible, assumption-free methodology, and does not need expertise on statistics, it has been used for the reliable data processing and then interpretations in the current paper. The GA, as general optimal clustering algorithm, makes the prediction process possible for many different patterns based on the existing data of interest by discovering the relations between the inputs (information) and outputs (responses).

## 2. Materials and Methods

### 2.1. Study Design

**Figure 1:**
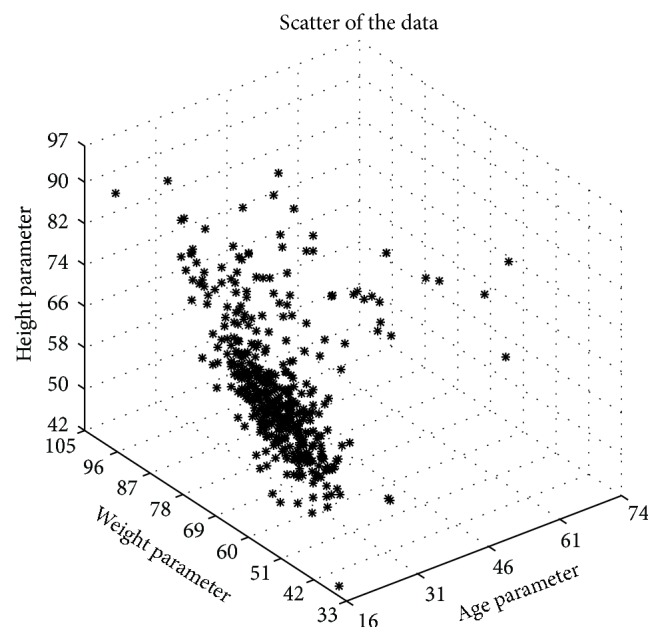
Scatter plot of the data consisted of age, weight, and height parameters.

In this study, dataset for healthy subjects was provided from the work of Sari and Cetiner [4]. The data includes measurement of age, weight, and height information of 484 volunteers. The age, weight, and height values of each subject are displayed in Figure 1.

In the data, tibial rotation values of each subject consisting of 4 components were given as right tibial external rotation (RTER), right tibial internal rotation (RTIR), left tibial external rotation (LTER), and left tibial internal rotation (LTIR). The rotation values were divided into 3 types as Type 1, Type 2, and Type 3 according to whether they were pathological or not, as seen in Table 1. Values between 0 and 20 degrees and between 65 and 90 degrees are accepted to be pathological. Values between 20 and 65 degrees are considered to be nonpathological [33, 34]. All types were divided into three clusters as Cluster 1, Cluster 2, and Cluster 3, based on the distribution of the data. This clustering was done according to age, weight, and height parameters as shown in Table 2. For all these rotation values, the number of subjects of the clusters in all types is also shown in Table 3.

The pragmatic aim of this paper is to predict pathological subjects from a population through various physical information (age, weight, and height) using the GA. As the GA clustering is of the mentioned advantages like flexibility and no need for assumption, it has been preferred for the trustworthy data processing in this study. Additionally, the KM clustering algorithm has also been used to decide which one is better in the prediction. Thence, this study keeps the light on capability of the GA in predicting pathological subjects based on the existing data by exploring the links between the inputs and outputs. Since the GA has been implemented for the first time for clustering in the prediction of subjects that they are either pathological or not, this study is believed to be a very significant contribution.

### 2.2. Genetic Algorithm

**Figure 2:**
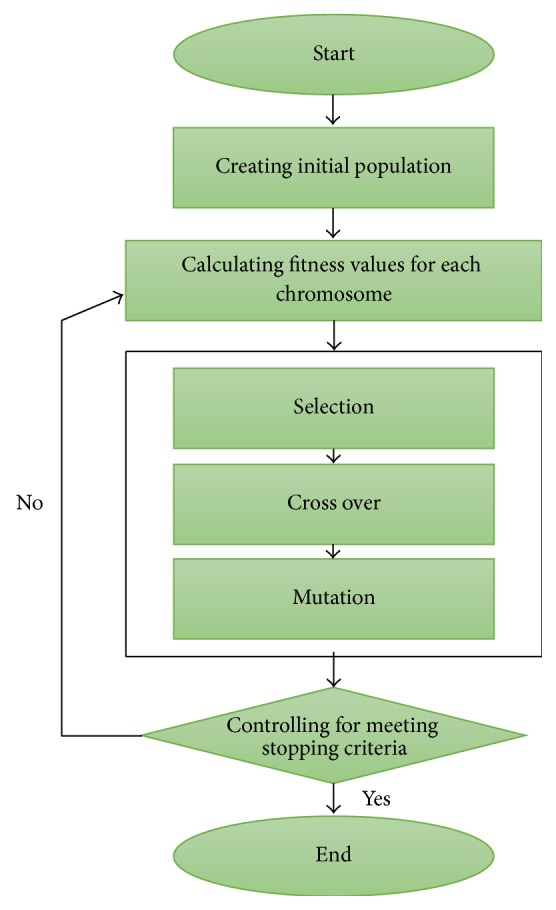
Flow diagram of the GA.

Darwin's theory of evolution has been a source of inspiration for many researchers in various disciplines. Many evolutionary algorithms have been developed using fundamental terms such as gene, natural selection, crossover, and mutation that Darwin put forward in his theory. One of the most important of these evolutionary algorithms is genetic algorithms (GAs). First, Goldberg and Holland [35] put the evolution process into a computer environment and took a step for the GAs. Goldberg [36] proved that the GAs have more than 80 examples in real life. Later, in terms of all those progresses, Koza [37] developed genetic programming. The main aim of the GA is that the strong individual survives and the weak die. The basic stages of determining the strong and weak individual are natural selection, crossover, and mutation. In the GA, it is aimed to find the best individual after individuals have passed through those stages. The flow diagram of the GA can be shown in Figure 2. The following subsections consist of the main steps of the GA.

#### 2.2.1. Initial Population

**Figure 3:**
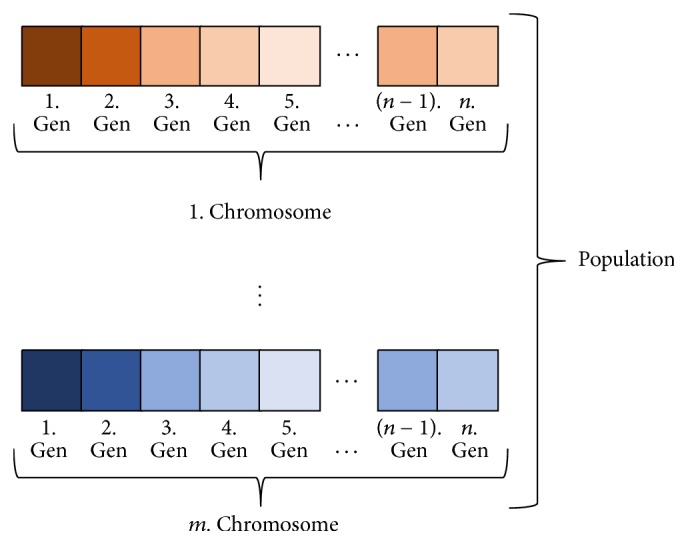
The display of gene, chromosome, and population.

For the solution space, random chromosomes with genes are created. The number of chromosomes generated for the solution indicates the size of the population. For example, the cluster of *m* chromosomes with randomly generated *n* genes to determine the maximization or minimization of a function is the initial population of the GA as explained in Figure 3. The values of all chromosomes in the fitness function of the problem are calculated. It has then been decided that if the individuals are strong or weak. The gene, chromosome, and population are illustrated in Figure 3.

#### 2.2.2. Selection

This step is the first step in which the principle of survival of the strong one begins to be implemented. At this stage, individuals are created to match each other in the future. The strongest candidates are determined according to the fitness values. According to the purpose of this algorithm, these candidates match each other and produce the highest quality of the generation. At the simplest level, if the problem is maximization, the individuals with the greatest fitness value are taken. Conversely, if the problem is minimization, this time and the individuals with the smallest fitness value are taken. The population of these individuals is called the transition population.

#### 2.2.3. Crossover

**Figure 4:**
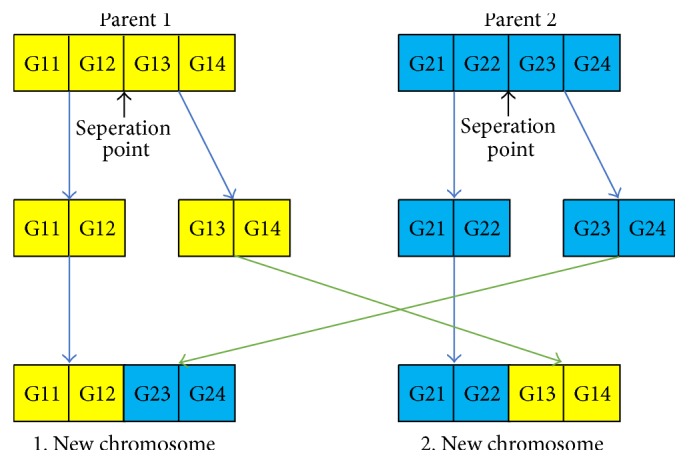
Sample of a crossover.

At this stage, a new generation is produced. High-quality individuals selected from natural selection are considered as parents and these individuals are matched to create new individuals. This mapping is created by replacing each individual gene sequence in each individual chromosome with each other. This process is called crossover. As an example, the second genes of Chromosome 1 and Chromosome 2 which have 4 genes will be matched and new individuals will be produced. This matching is illustrated in Figure 4.

#### 2.2.4. Mutation

**Figure 5:**
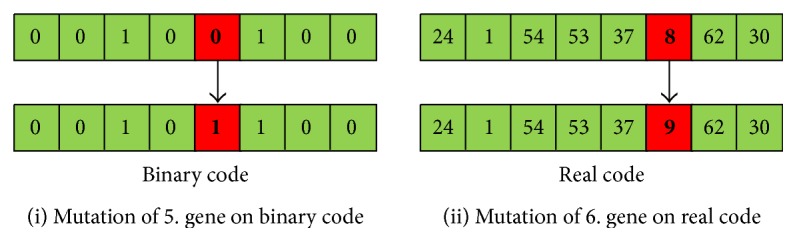
Examples of mutation on binary code and real code.

Sometimes, some genes may remain the same even if matching has repeatedly been carried out in the individuals to be matched. This situation prevents the formation of different individuals. So, it may not deliver the best solution. Although the probability of occurrence of this situation is very low, to prevent problems due to this situation, a very small change can be made in a gene of the created individuals. Thus, different individuals occur and future generations also become different. Two examples of mutations are shown in Figure 5.

As can be seen from the figure, the mutations made in the binary codes are a general reverse translation process. This converts 0 to 1 or 1 to 0. This means that mutations in binary code can make a big difference in terms of gene diversity. When looking at real coded chromosomes, very small changes are made in the genes, depending on their value. The effect obtained with very small spins in the real code is equivalent to the large effect in the binary code.

 Creating initial population, selecting strong individuals from this population (natural selection process), and creating high-quality generation by matching these strong individuals each other (crossover), the process of eliminating the problem of producing the same generation from similar genes (mutation) is repeated in each iteration. It is aimed at producing a better generation as a result of each iteration. When the specified number of iterations is reached, the algorithm is terminated and the optimum value is found.

The GA does not circulate at all points in solution space. In all steps, it cannot travel every point because it has randomness as in nature. The GA tries to predict the best by improving the randomly determined population. More details on the GA can be found, for instance, in [38–42].

The GA have been implemented for solving problems in many fields ranging from medical applications [43, 44] to prediction of heavy rainfall based on certain medical parameters [45]. However, the prediction of tibial rotation types using the GA is new. This article makes a thorough study of some physical information and examines their relationship with the tibial motion factors based on the GA. The pseudocode of the GA has been presented as shown in Pseudocode 1.

#### 2.2.5. Genetic Algorithm (GA) Clustering

The GA investigates for the optimal solution together with its own processes like selection, crossover, and mutation. For clustering, the optimum solution is searched as many as the number of clusters. The distance is based on those optimum solutions. The optimum solutions are then considered to be cluster centers. The issue of finding center required in clustering algorithms is sorted out by using the GA. Although one encounters various GA clustering examples in the literature for different problems [46–51], to the best knowledge of the authors, for the first time, the GA has been implemented to estimate pathological subjects through various physical parameters.

### 2.3. *K*-Means Clustering Algorithm

The *K*-Means (KM) clustering algorithm is one of the fastest, simplest, and most common methods in clustering problems. Firstly, the KM was discovered by MacQueen [52]. The way that the algorithm works is given as follows. The algorithm divides *N* data into *k* groups according to their distance to each other. The algorithm aims to find the best cluster center for each iteration. Cluster centers are updated for each iteration. This is done by taking the average of the new cluster center and the old cluster centers. The name of the algorithm stems from this procedure.

As clustering-based algorithm is based on the points that are the closest to each other, an objective function must be already given in the KM approach and thus the problem will be a minimization problem. The Euclidean distance is used in the algorithm as follows [53]:

(1) $$\begin{array}{c} D=\overset{K}{\underset{j=1\;}{\sum }}\overset{N}{\underset{i=1}{\sum }}{\left\|\;x_{i}-C_{j}\;\right\|}^{2}, \end{array}$$

where *x*_*i*_, 1 ≤ *i* ≤ *N*, and *C*_*j*_, 1 ≤ *j* ≤ *K*, stand for set of *N* data and set of cluster centroids, respectively. The distance between any two *p*-dimensional patterns *X*_*i*_ and *X*_*j*_ can be expressed as follows [54]:

(2) $$\begin{array}{c} d\left(\;X_{i},X_{j}\;\right)=\sqrt{\overset{p}{\underset{m=1}{\sum }}{\left(X_{im}-X_{jm}\right)}^{2}}. \end{array}$$

## 3. Results and Discussion

In this study, each one of all rotation values RTER, RTIR, LTER, and LTIR is divided into three types as Type 1, Type 2, and Type 3. For all types, success of Cluster 1, Cluster 2, and Cluster 3 has been observed.

For example, Type 1 values for RTER are 0, 17, and 22 for Cluster 1, Cluster 2, and Cluster 3, respectively. So, there are 39 subjects in total. These are 0.00%, 43.59%, 56.41%, respectively, as the percentage values from Table 4. Taking these values into consideration, if we look at the results of the KM algorithm in Table 6, Type 1 value for RTER is 39, and these values are 0, 2, and 37 for Cluster 1, Cluster 2, and Cluster 3, respectively. Even for this situation, the failure of the KM for Type 1 can be seen. Looking at the percentage will give a clearer interpretation. It is 0.00%, 5.13%, and 94.87%, respectively. By comparing the results of the KM and actual values, the KM found these values as 5 and a percentage of 5.13%, while Type 1 has a real value of 17 and a percentage of 43.59% for Cluster 2. Likewise, if the same assessment is made for Cluster 3, the ratio should be 56.41%, which is 94.87%. It can be simply assessed as follows: the KM has found it to be 43.59%, even though the actual rate is 5.13%. If proportional, the KM will achieve an accuracy rate of 8.49%.

If all these evaluations are done for the GA by considering RTER again, the GA has found them to be 0, 17, and 22 that real values of Cluster 1, Cluster 2, and Cluster 3 for Type 2 are 0, 17, and 22, respectively. So, that is 100.00% success as seen from Table 5.

As in all optimization algorithms, the GA requires large number of elements to be able to produce accurate results. The real value of RTIR-Type 2 is 423. From these data, 48 subjects belong to Cluster 1, 223 subjects belong to Cluster 2, and 152 subjects belong to Cluster 3. In percent, Cluster 1, Cluster 2, and Cluster 3 are 11.35%, 52.72%, and 35.93%, respectively. The KM has produced these values as 36, 263, and 124; in percent, they are as follows: 8.51%, 62.18%, and 29.31%. The real RTIR-Type 2 has Cluster 1 value of 48 and a KM value of 36. It has been found to be 8.51%, while the real one is 11.35%, with the accuracy rate of 74.98. Yet, the KM has been found to be 263 (62.18%) and 124 (29.31%) for Cluster 2 and Cluster 3, respectively. Again, to evaluate the accuracy percentage, the real Cluster 2 value is 52.72% while the KM is found to be 62.18%. This is of accuracy rate 84.79%. In the same way, the real value of Cluster 3 is 35.93% while the value for the KM is 29.31%. Again, the accuracy rate is 81.58%.

If the same considerations are made for the GA, the RTIR-Type 2 values have been found to be 59, 226, and 138 for Cluster 1, Cluster 2, and Cluster 3, respectively. The produced values of the GA for the clusters are 13.95%, 53.43%, and 32.62%, respectively. As seen in Table 4, the actual values for the three clusters are 11.35%, 52.72%, and 35.93%, respectively. The accuracy rates calculated in the GA are 81.36%, 98.67%, and 90.79%.

If all values are recovered, for the GA, accuracy rate of Cluster 1 for RTIR-Type 2 is 81.36% while it is 74.98% for the KM for the same parameters (see Table 7). Likewise, for the GA, accuracy rate of Cluster 2 for RTIR-Type 2 is 98.67% while it is 84.79% for the KM for the same factors. Finally, for the GA, accuracy rate of Cluster 3 of RTIR-Type 2 is 90.79% while the KM produced is 81.58% for the same parameters. As can be seen from these values, success of the clustering of RTIR-Type 2 of the GA is much higher in comparison with success of the KM. Especially for Cluster 2, which has the highest number of subjects, the GA is leading by a huge difference. The reason for this is that increasing the number of subjects leads to increasing the success. Note that, in general, in case of large of number of subjects, the GA is found to be far more successful than the KM clustering for the current problem.

The accuracy rates are compared in Table 7 to show which algorithm is more successful than the other. When these ratios are calculated, firstly, the values in Table 4 are compared with Table 6 and written in the KM column in Table 7. Likewise, the values in Table 4 are compared to Table 5 and written in the GA column.

As an example, in Table 4, the real ratio value of Cluster 2 for LTER-Type 2 is 44.82%. The same value is found to be 67.79% for the KM in Table 6. The accuracy rate of LTER-Type 2-Cluster 2 is obtained as 66.12% as seen in Table 7. If the same operations are performed for the GA in Table 5, this value is 45.10%. If these values are compared, a success of 99.38% is achieved by the GA. Table 7 has been generated by repeating the same procedures for all rotation values. As can be seen from Table 7, the GA is mostly clustering much more successfully than the KM algorithm.

For a long time, the GA has been used as a very powerful algorithm in various problems of science. To the best knowledge of the authors, in the current paper the GA has been applied to the tibial rotation for the first time. It was tested if it would be successful in the field as is the case in a large kind of problems. The GA has been seen to produce very effective results in predicting the tibial rotation types through the physical information. The application to the current problem helps health providers to predict the type of the rotation, that is, pathological or nonpathological.

Clustering success was targeted by dividing each one of the rotation values RTER, RTIR, LTER, and LTIR into pathological (Type 1 and Type 3) or nonpathological (Type 2) classes. In the present problem, the number of clusters for the genetic algorithm is given by the user. Subjects are divided into 3 clusters (Cluster 1, Cluster 2, and Cluster 3) by considering age and weight parameters. Taking into consideration these values, the effect of physical information on the tibial rotations has been investigated. Then the results of the GA have been compared with the results of the KM clustering algorithm. In case of large of number of subjects, it has strikingly been seen that the GA has been found to be far more effective than the KM clustering algorithm for optimizing correctly the current tibial problem. It is noticeable that the dataset is consisting of subjects mostly younger than 30 years old; the current study may not be very decisive enough for that subjects who are older than 30.

## 4. Conclusion and Further Research

This paper has predicted pathological subjects from a population through various physical information using the genetic algorithm. Unlike traditional approaches, the GA has thus accomplished to predict the types of the tibial rotation through several physical factors: age, weight, and height. Since the real values of each rotation type are known, the results of both the GA and the KM clustering algorithm are compared with these actual values. The clustering with the GA has been done for the first time in the prediction of tibial rotations. The simulation results have proven the superiority of the GA over the other competitive counterpart, the KM clustering algorithm. The GA has been seen to be very successful on optimizing the tibial rotation data assessments with many subjects even though the KM algorithm has similar effect with the GA in clustering with a small number of subjects. It has been concluded that findings are clinically expected to be very useful for health providers in organizing proper treatment programs for patients. For future research, this study could be divided into more clusters depending on the structure of the data but the structure of the current dataset is limited to have more clusters from medical point of view. In the forthcoming works, more clusterable and thus more illustrative results may be found with various datasets.

## Figures and Tables

**Pseudocode 1:**
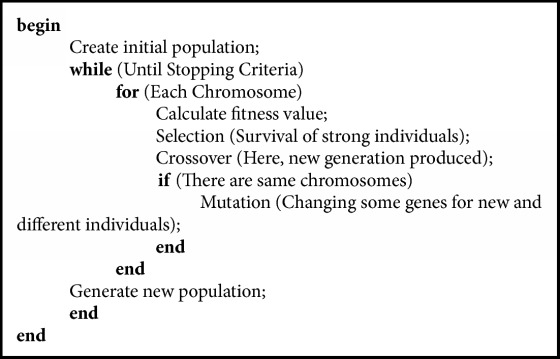
Pseudocode of the genetic algorithm.

**Table 1:** Type values of each rotation and number of subjects.

	RTER	RTIR	LTER	LTIR
Type 1 (≤20°)	39	33	37	51
Type 2 (20°–65°)	391	423	357	414
Type 3 (>65°)	24	28	90	19

**Table 2:** Clusters and number of subjects.

	Age	Weight	Height	Number of subjects
Cluster 1	>30	-	-	52
Cluster 2	≤ 30	≤ 60	≤ 1.70	249
Cluster 3	≤ 30	>60	>1.70	183

**Table 3:** Number of types in each cluster for every rotation type.

		Cluster 1	Cluster 2	Cluster 3	Total
RTER	Type 1	0	17	22	39
	Type 2	50	183	158	391
	Type 3	2	49	3	24
	Total	52	249	183	484

RTIR	Type 1	1	7	25	33
	Type 2	48	223	152	423
	Type 3	3	19	6	28
	Total	52	249	183	484

LTER	Type 1	1	16	20	37
	Type 2	47	160	150	357
	Type 3	4	73	13	90
	Total	52	249	183	484

LTIR	Type 1	3	14	34	51
	Type 2	49	218	147	414
	Type 3	0	17	2	19
	Total	52	249	183	484

**Table 4:** Real cluster values and percentages of all tibial rotation types.

Real									
		Cluster 1	Percent (%)	Cluster 2	Percent (%)	Cluster 3	Percent (%)	Total	Percent (%)
RTER	Type 1	0	*-*	17	*43.59*	22	*56.41*	39	*100.00*
	Type 2	50	*12.79*	183	*46.80*	158	*40.41*	391	*100.00*
	Type 3	2	*3.70*	49	*90.74*	3	*5.56*	54	*100.00*

RTIR	Type 1	1	*3.03*	7	*21.21*	25	*75.76*	33	*100.00*
	Type 2	48	*11.35*	223	*52.72*	152	*35.93*	423	*100.00*
	Type 3	3	*10.71*	19	*67.86*	6	*21.43*	28	*100.00*

LTER	Type 1	1	*2.70*	16	*43.24*	20	*54.06*	37	*100.00*
	Type 2	47	*13.17*	160	*44.82*	150	*42.01*	357	*100.00*
	Type 3	4	*4.44*	73	*81.11*	13	*14.45*	90	*100.00*

LTIR	Type 1	3	*5.88*	14	*27.45*	34	*66.67*	51	*100.00*
	Type 2	49	*11.84*	218	*52.66*	147	*35.50*	414	*100.00*
	Type 3	0	*-*	17	*89.47*	2	*10.53*	19	*100.00*

**Table 5:** Results of the GA for all tibial rotation types.

GA									
		Cluster 1	Percent (%)	Cluster 2	Percent (%)	Cluster 3	Percent (%)	Total	Percent (%)
RTER	Type 1	0	*-*	17	*43.59*	22	*56.41*	39	*100.00*
	Type 2	30	*7.67*	205	*52.43*	156	*39.90*	391	*100.00*
	Type 3	1	*1.85*	48	*88.89*	5	*9.26*	54	*100.00*

RTIR	Type 1	1	*3.03*	2	*6.06*	30	*90.91*	33	*100.00*
	Type 2	59	*13.95*	226	*53.43*	138	*32.62*	423	*100.00*
	Type 3	2	*7.14*	21	*75.00*	5	*17.86*	28	*100.00*

LTER	Type 1	1	*2.70*	13	*35.14*	23	*62.16*	37	*100.00*
	Type 2	38	*10.64*	161	*45.10*	158	*44.26*	357	*100.00*
	Type 3	1	*1.11*	75	*83.33*	14	*15.56*	90	*100.00*

LTIR	Type 1	1	*1.96*	17	*33.33*	33	*64.71*	51	*100.00*
	Type 2	9	*2.17*	222	*53.62*	183	*44.20*	414	*100.00*
	Type 3	0	*-*	17	*89.47*	2	*10.53*	19	*100.00*

**Table 6:** Results of the KM clustering for all tibial rotation types.

KM									
		Cluster 1	Percent (%)	Cluster 2	Percent (%)	Cluster 3	Percent (%)	Total	Percent (%)
RTER	Type 1	0	*-*	2	*5.13*	37	*94.87*	39	*100.00*
	Type 2	42	*10.74*	207	*52.94*	142	*36.32*	391	*100.00*
	Type 3	4	*7.41*	43	*79.63*	7	*12.96*	54	*100.00*

RTIR	Type 1	0	*-*	1	*3.03*	32	*96.97*	33	*100.00*
	Type 2	36	*8.51*	263	*62.18*	124	*29.31*	423	*100.00*
	Type 3	2	*7.14*	23	*82.14*	3	*10.72*	28	*100.00*

LTER	Type 1	1	*2.70*	1	*2.70*	35	*94.60*	37	*100.00*
	Type 2	36	*10.08*	242	*67.79*	79	*22.13*	357	*100.00*
	Type 3	2	*2.22*	67	*74.45*	21	*23.33*	90	*100.00*

LTIR	Type 1	1	*1.96*	3	*5.88*	47	*92.16*	51	*100.00*
	Type 2	36	*8.70*	251	*60.63*	127	*30.67*	414	*100.00*
	Type 3	0	*-*	19	*100.00*	0	*-*	19	*100.00*

**Table 7:** Comparison of the GA and the KM rates.

		Cluster 1		Cluster 2		Cluster 3	
		GA	KM	GA	KM	GA	KM
RTER	Type 1	-	-	**100.00**	11.77	**100.00**	59.46
	Type 2	59.97	**83.97**	**89.26**	88.40	**98.74**	89.88
	Type 3	50.00	50.00	**97.96**	87.76	**60.04**	42.90

RTIR	Type 1	**100.00**	-	**28.57**	14.29	**83.34**	78.13
	Type 2	**81.36**	74.98	**98.67**	84.79	**90.79**	81.58
	Type 3	66.67	66.67	**90.48**	82.62	**83.34**	50.02

LTER	Type 1	**100.00**	100.00	**81.27**	6.24	**86.97**	57.15
	Type 2	**80.79**	76.54	**99.38**	66.12	**94.92**	52.68
	Type 3	25.00	**50.00**	**97.34**	91.79	**92.87**	61.94

LTIR	Type 1	33.33	33.33	**82.36**	21.42	**97.06**	72.34
	Type 2	18.33	**73.48**	**98.21**	86.85	80.32	**86.39**
	Type 3	-	-	**100.00**	89.47	**100.00**	-
